# Hospital Meetings

**Published:** 1901-06-29

**Authors:** 


### HOSPITAL MEETINGS.

### CENTRAL LONDON THROAT, NOSE, AND EAR HOSPITAL.

Captain Hutton, the chairman of the committee, presided at the twenty-eighth annual meeting of the Central London Throat, Nose, and Ear Hospital, Gray's Inn Road, on the 12th inst. The report presented stated that there had been during the year 1900 8,428 out-patients with 53,041 attendances. The majority of the patients were between the ages of 20 and 40, and of these 60 per cent. were males—representing the breadwinner at his best period of wage-earning life. No less than 1,560—equal to 20 per cent.—of the patients were sent direct from medical practitioners—a demonstration of the appreciation of the value of the hospital treatment by those best qualified to judge. In this way not only were the patients receiving benefit but the correspondence between the staff and medical men outside constituted a consultation, and the hospital became the centre for the dissemination of the special knowledge acquired of their particular branch of medical science. Two hundred and forty in-patients were admitted to the wards; this number being the smallest on record since 1891. This diminution arose from the circumstance that cases formerly regarded as beyond treatment were now received, and with the most beneficial results. Consequent on this fewer beds were available, since the small wards, containing two beds, had often to be set apart for the nursing of one patient only. In that department also many patients requiring prompt surgical treatment were sent in by their family medical attendant. The ordinary income was £2,032, as compared with £2,474 in the previous year. To this is added an extraordinary item of £915, the proceeds of an entertainment at the Queen's Hall in November, when Mr. W. T. Maud, special artist to the "Graphic," gave a lecture, entitled "Four Months in Beleaguered Ladysmith," Sir Redvers Buller presiding. The ordinary expenditure was £2,212, and the extraordinary expenditure £116. Of this last £90 was interest to the bankers on the loan, and £26 for building improvement. During the year the liabilities had been reduced from £4,310 to £3,579, while the assets had been augmented, cash in hand having increased from £260 to £1,047.

Mr. Lennox Browne, in proposing the adoption of the report, mentioned that the Prince of Wales's Fund had given £150 towards the erection of an operating-room, which was much needed, since though their tables of mortality were at least as low as those of any other hospital, yet the days of convalescence might be reduced by the use of the most complete methods of sterilisation and so forth.

The report was adopted, the committees, chairman and vice-chairman re-elected, and cordial votes of thanks accorded to the medical officers and to the chairman. The proceedings then terminated.

